# Prevalence and capsular type distribution of group B *Streptococcus* isolated from vagina of pregnant women in Nghe An province, Vietnam

**Published:** 2020-02

**Authors:** Tran Quang Hanh, Vu Van Du, Pham Thu Hien, Duong Dinh Chinh, Cao Ba Loi, Nguyen Manh Dung, Do Ngoc Anh, Tran Thi Kieu Anh

**Affiliations:** 1 Department of Obstetrics, Nghe An Obstetrics and Pediatrics Hospital, Nghe An, Vietnam; 2 Department of Treatment on Demand, National Hospital of Obstetrics and Gynecology, Hanoi, Vietnam; 3 Department of Treatment on Demand, National Hospital of Pediatrics, Hanoi, Vietnam; 4 Department of Neurology, Nghe An Friendship General Hospital, Nghe An, Vietnam; 5 Department of Science and Training, National Institute of Malaria Parasitology and Entomology, Hanoi, Vietnam; 6 Department of Anesthesiology and Critical Care, 108 Military Centre Hospital, Hanoi, Vietnam; 7 Department of Medical Parasitology, Military Medical University, Hanoi, Vietnam; 8 Department of Paediatric, Vinh Medical University, Nghe An, Vietnam

**Keywords:** Group B *Streptococcus*, Prevalence, Serotypes, Nghe An, Vietnam

## Abstract

**Background and Objectives:**

Identification of GBS serotypes provides helpful information for appropriate the development of suitable vaccines; however, no reports from Vietnam have been published. This study has been performed to find the prevalence and serotypes of group B *Streptococcus* isolated from vagina of pregnant women in Nghe An province, Vietnam.

**Materials and Methods:**

Vaginal swabs were collected from pregnant women at 35–37 weeks of gestation at the Nghe An Obstetrics and Pediatrics Hospital, Vietnam between May 2018 and July 2019. The swabs were cultured on 5% sheep blood agar for isolation of GBS. All isolates were identified using the Gram staining, CAMP test and specific PCR. GBS strains were serotyped using the multiplex PCR assays.

**Results:**

The prevalence of vaginal GBS colonization was 9.20% of 750 participants. Among the isolates, serotypes III (39.13%) and V (31.89%) were the most frequent, followed by serotypes Ia (11.59%), VI (11.59%), Ib (2.90%), II (1.45%) and VII (1.45%), respectively. Serotypes IV, VIII and IX were not found.

**Conclusion:**

The prevalence of GBS in the Nghe An province of central Vietnam was similar to reports from other parts of the world. The predominat GBS serotypes (III, V, Ia and VI) were slightly different from those previously described from other regions around the world. The high frequency of serotype VI was a notable feature of the strains from pregnant women in Vietnam.

## INTRODUCTION

*Streptococcus agalactiae* (group B *Streptococcus*, GBS) is a Gram-positive bacterium that is an important neonatal pathogen of severe and invasive neonatal infections such as sepsis and meningitis, which are associated with high morbidity and mortality rates (1–4). This etiological agent also occurs as an invasive infections in immunocompromised patients and elderly persons (5–7). In pregnant women, GBS has been found in 10–30%, usually without any symptoms and are at risk of transmitting it to their newborn babies (6, 8, 9). In newborns, invasive infections due to GBS are associated with a mortality rate of 4% to 6% (9). While GBS infections virtually never cause maternal death, about 10–60% of them result in miscarriage or stillbirth (6, 10). The prevalence of invasive GBS infections are gradually increasing worldwide, particularly in older adults with underlying diseases, such as diabetes, cardiovascular disease, and cancer (7, 11). Therefore, the prevention and treatment of these infections are growing in importance (7, 11, 12).

The GBS are currently divided into ten serotypes based on the antigenicity of their capsular polysaccharides (CPS) and are categorized as Ia, Ib, II, III, IV, V, VI, VII, VIII and IX (13, 14). Epidemiological surveys around the world have indicated that distribution of GBS serotypes are geographically different (6, 7). According to previous studies, serotype III is the most predominant, followed by Ia, Ib, II and V (6, 15, 16). These CPS is an important virulence factor of GBS (6), (17) and of there, serotypes Ia, III and V are reported to account for the majority of invasive cases of GBS (18). Therefore, routine screening for maternal colonization by GBS should be performed for prevention of neonatal infections (9, 16). According to Africa et al. (2018), an understanding of the serotype prevalence associated with GBS colonization and invasive disease in newborns is necessary to inform the development of suitable vaccines (18).

Identification of GBS serotypes, based on immunodiffusion tests and commercial latex agglutination (LA) methods, are the most widely used but these tests are only moderately reliable, resulting in nontypeability (NT) or erroneous serotyping of the isolates (2, 5). Because of the limitations of immunological methods, several molecular approaches have been developed for the differentiation of GBS serotypes (5).

Previously, there are many publications from Asian countries as China (15, 16), Japan (7), Korea (19), Thailand (18, 20) and Philippines (20) but very limited data is available on the serotypes distribution of GBS in Vietnam. Thus, the aim of this study was to determine the prevalence and serotypes of GBS isolated from vagina of pregnant women in Nghe An province, Vietnam.

## MATERIALS AND METHODS

**Clinical isolates and identification of GBS.** The present cross-sectional study was conducted between May 2018 and July 2019. Vaginal samples was obtained from vagina of 750 healthy pregnant women at 35–37 weeks of gestation were evaluated at the Nghe An Obstetrics and Pediatrics Hospital (500 beds, Nghe An province, Vietnam). Vaginal specimens were taken from each patient’s vagina by trained nurses with sterile cotton swabs. After that, vaginal swabs were transported to the clinical microbiology laboratory within 2 hours for isolation of GBS. Samples were inoculated onto blood agar containing 5% sheep blood (Himedia, India) and incubated at 37 °C for 24 h under 5% carbon dioxide (CO_2_) atmosphere. The samples that no growth after 24 hours were incubated for a further 24 hours before being declared as culture-negative. In order to isolate GBS from vagina cultures-positive, colonies of suspected GBS were subcultured onto a sheep blood agar plate (Himedia, India) and was incubated at 37 °C for 18 to 24 hours under 5% CO_2_ atmosphere. Colonies on blood agar plates were first confirmed as GBS using Gram staining and CAMP test. After the initial morphological identification, GBS isolates were identified by PCR and sequencing.

**Genomic DNA isolation.** DNA of GBS was extracted from isolates using QIAamp DNA Mini Kit (Cat. No51304, QIAGEN, Hilden, Germany), following manufacture recommendation. After that quality and quantity of extracted DNA was estimated using a NanoDrop ^TM^ 2000 Spectrophotometer at 260 nm (Thermo Fisher Scientific, USA). Extracted DNA was diluted in double distilled water and maintained at −20 °C until used in the PCR.

**Molecular identification of GBS strains.** GBS isolates was confirmed by molecular techniques using the specific primer pair of *dlt*S-F (5′-AGG AAT ACC AGG CGA TGA ACC GAT-3′) and *dlt*S-R (5′-TGC TCT AAT TCT CCC CTT ATG GC-3′) (Integrated DNA Technologies, USA) for the *dlt*S gene (2). The components of PCR reaction were as follows: 25 μl of 2× PCR SuperMix (Quantabio, USA), 1 μM each primer with final concentration of 10 pmol, 5 μl of template DNA and molecular grade distilled water up to 50 μl. The conditions were an initial denaturation step at 95 °C for 5 min, followed by 35 cycles (95 °C for 60 seconds, 55 °C for 60 seconds, and 72 °C for 60 seconds), with a final extension at 72 °C for 10 min. Sterile deionizer water used as negative control.

**Determination of GBS serotypes based on multiplex PCR technique.** Serotypes of GBS were identified by multiplex PCR assays using specific primers (Integrated DNA Technologies, USA) as described by Poyart et al. (2007) (2). The capsular types were collected on three groups of annealing temperature as follows: Types 1a, 1b, II and III at 58 °C; IV and V at 59 °C; VI, VII and VIII at 56 °C. Total volume of multiplex PCR reactions was 20 μl containing 2 μl of DNA solution, 10 μl 2× SuperMix (Quantabio, USA), 0.5 μl of each primer (0.25 μM) and distilled water up to 20 μl. PCR amplification was carried out with Thermo Mastercycler Gradient (Thermo Fisher Scientific, USA). The conditions for the multiplex PCR assays were as follows: an initial denaturation step at 95 °C for 5 minutes, followed by 35 cycles of denaturation at 94 °C for 60 seconds, annealing at the respective annealing temperature for 60 seconds and extension at 72 °C for 60 seconds and a final extension of 72 °C for 10 minutes.

The PCR and multiplex PCR products were analyzed on 1.5% agarose gels containing 0.5 μg/ml ethidiumbromide in 1× TBE buffer for about 1.5 h at 90V and visualized with UV illumination (UVP, Canada). The PCR product sizes was determined by a 50 bp (Thermofisher, USA) and 100 bp size marker (Norgen, Canada).

**16S rRNA gene sequencing.** Amplification of the 16S rDNA gene were achieved with the primers 27F (5′-AGAGTTTGATCCTGGCTCAG-3′) and 1492R (5′-GGT TAC CTT GTT ACG ACT T-3′) (21). The PCR products were purified with a GeneJET PCR purification Kit (#K0701, Thermo Fisher Scientific, USA). After that PCR products of 16S gene from six isolates were sent to First BASE Laboratories Sdn-Bhd service (Kembangan 43300, Selangor, Malaysia) for automatic sequencing in both directions, using the same primers which were used in the PCR. The sequence accuracy of data was confirmed by two-directional sequencing. The GenBank/EMBL accession numbers of the sequences derived from strains GBS20, GBS23, GBS25, GBS28, GBS29 and GBS31 are MK942595, MK942596, MK942597, MK942598, MK942599 and MK942600, respectively.

**Statistical analyses.** Statistical analyses were performed using IBM SPSS Statistics for Windows, version 20.0 (IBM Corp., Armonk, NY, USA). The sequences of the 16S rDNA regions of GBS were analyzed independently by comparing with related sequences available in the GenBank database, using BLAST guidelines (https://blast.ncbi.nlm.nih.gov/Blast.cgi).

**Ethics approval and consent to participate.** The purpose and benefits of the study were informed to the patients. The inclusion criteria were pregnant women at 35–37 weeks of gestation: (i) agreed to participate in the study, (ii) signed a written informed consent, did not take antibiotics within 2 weeks and (iii) gave informed consent were eventually included in the study. The study protocol was approved by the Scientific and Ethical Committee at the National Institute of Malariology Parasitology and Entomology (Ha Noi, Vietnam) in March 2018 (ethics code: 264/QĐ-VSR).

## RESULTS

**Fig. 1.**
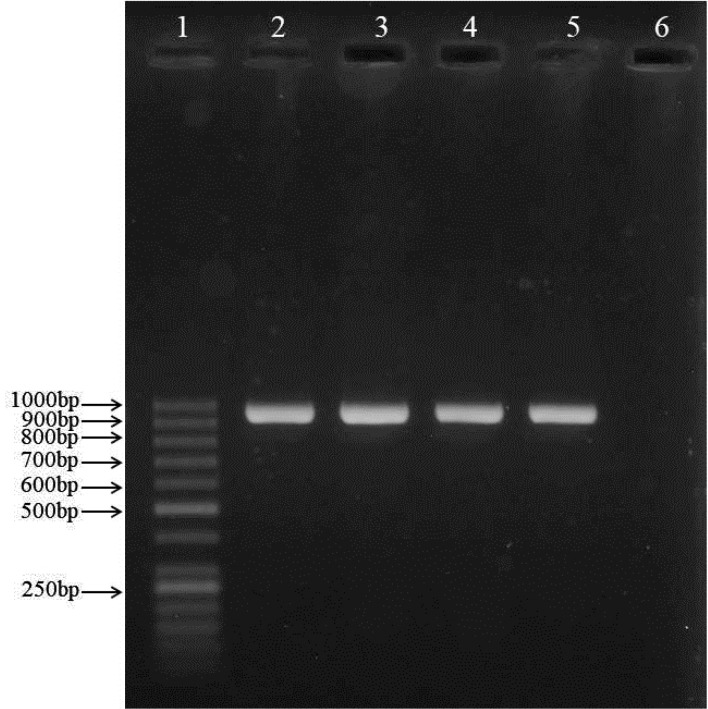
Gel electrophoresis of GBS-specific PCR products targeting the 952 bp *dlt*S gene Lane 1: molecular size standard (50 bp DNA ladder); lanes 2–5 (strain GBS24 to GBS27): clinical GBS samples; lane 6: negative control

The 750 vaginal samples were collected from vagina of pregnant women at 35–37 weeks of gestation between May 2018 and July 2019. In overall, 69 (9.20%) were found to be positive for GBS by culture, Gram staining, CAMP test. All of the isolates which were GBS culture-positive, were also positive by species-specific PCR primers (Fig. 1). We also deposited six sequences of the 16S rDNA regions of GBS in the NCBI database (GenBank, USA) under accession number MK942595 to MK942600.

**Fig. 2.**
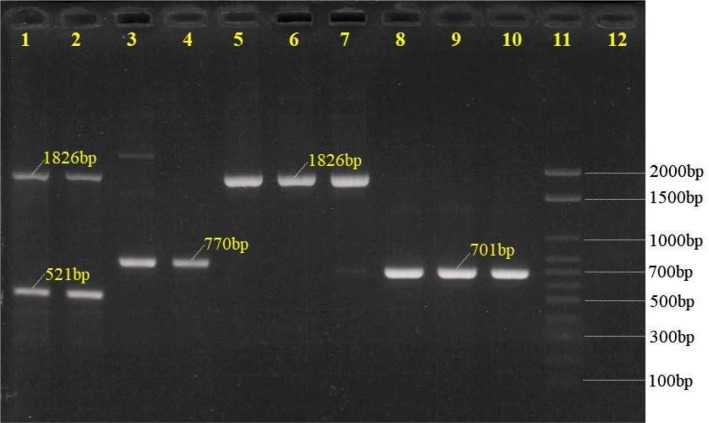
The multiplex PCR patterns of serotypes Ia, Ib, III (reaction 1) and V (reaction 2). Lanes 1 and 2 denoted to those of serotype Ia; lanes 3 and 4 denoted to those of serotype Ib; lanes 5–7 denoted to those of serotype III, lanes 8–10 denoted to those of serotype V; lane 11: 100bp ladder molecular weight marker; lane 12: negative control.

By multiplex PCR method, seven of the ten currently recognized GBS serotypes were identified, the most common serotype was III (n = 27, 39.13%) and V (n = 22, 31.89%), followed by Ia (n = 8, 11.59%), VI (n = 8, 11.59%), Ib (n = 2, 2.90%), II (n = 1, 1.45%) and VII (n = 1, 1.45%); the serotype distribution and frequency is shown in Table 1 and Fig. 2. Serotypes IV, VIII and IX were not detected. There were no significant correlations between age groups (< 30 and ≥ 30) and serotypes (p = 0.894).

**Table 1.** Frequency distribution of capsular types among pregnant women in Nghe An province, Vietnam

**Capsular type**	**Number**	**Frequencies (%)**
Ia	8	11.59
Ib	2	2.90
II	1	1.45
III	27	39.13
V	22	31.89
VI	8	11.59
VII	1	1.45
Total	69	100

## DISCUSSION

GBS is known to be the most common infectious cause of morbidity and mortality in neonates although preventive actions have decreased their incidence in many countries (4, 6). The results from different studies indicate that the prevalence of maternal GBS colonization, which is primary risk factor of neonatal GBS infections, differs in different countries (6, 18, 22). According to the CDC, routine screening for all pregnant women at 35–37 weeks gestation was necessary to prevent neonatal GBS infections (9, 16) but in Vietnam this issue has received very little attention. Therefore, the current study has been performed to determine the prevalence of GBS isolated from vagina of pregnant women in Nghe An province, Vietnam.

Multiple studies have demonstrated the prevalence of GBS among pregnant women varies between countries and different regions, ranging from 3% to 41% (9, 18, 23). In this study, GBS colonization rate among pregnant women in Nghe An province was found to be 9.20%. The results of our study was similar to that of previous researches in Iran (9.1%) (24), Eastern Asia (9.2%) (22), Turkey (9.2%) (25), and Korea (10.0%) (26). Lower GBS prevalence was reported from Cameroon (4.0%) (27), India (2.3%) (28), China (7.1%) (16). The higher prevalence rates reported in Namibia (13.6%) (6), Ethiopia (15.7%) (29), South Africa (16.6%) (18), Saudi Arabia (27.6%) (30). Studies from Brazil, Switzerland reported also higher prevalence of GBS among pregnant women than current study (31, 32). The prevalence of GBS in Vietnam are not very different from those in other parts of the world. Varying prevalence could be due to multiple factors including regional differences, sampling period, collection site of specimens, collection and culture method for GBS isolation and identification, etc. (18, 22, 33). Our finding is probably the first report of GBS infections in Vietnammese pregnant women. Therefore, more studies are required to determine the specific rate of GBS-vaginal colonization in Vietnam.

The capsular polysaccharide (CPS) represents an important virulence factor for most encapsulated streptococci, including GBS, and has been related to the bacterial disease clinical manifestations and invasiveness (34). Therefore, it is considered as one of the main targets in the investigation for development of an effective and safe GBS vaccine (18). Previous studies showed that the serotype distribution of GBS varies in different geographical regions and ethnic origin of pregnant women (18, 35). Nevertheless, there were no reports of GBS seretypes available in Vietnam. The present study aims to report the distribution of GBS serotypes among healthy pregnant women at 35–37 weeks of gestation in NgheAn province, Vietnam, using multiplex PCR.

Identification of GBS serotypes in clinical laboratory is becoming increasingly important since it is one of the most important virulence factors and antigenic determinant. In a systematic review or meta-analysis, Madrid et al. (2017) indicated that serotypes Ia, Ib, II, III and V account for 97% of invasive isolates in all geographical regions (36). Owing to its importance in GBS pathogenesis, capsular types is considered to be the prime vaccine candidate for development of a vaccine against GBS infections (17, 37). Our study detected GBS serotypes I–III and V–VII among pregnant women in Nghe An province but not IV, VIII and IX. The most common serotypes in this study were III accounting for 39.13% of 69 GBS infections in pregnant women, followed by V (n = 22, 31.89%), while serotypes Ia, Ib, II, VI and VII were represented in lower percentages, ranging from 1.45 to 11.59%. Notably, serotype VI has a relatively high prevalence (11.59%). These results of our study were slightly different from reports from around the world. Serotypes Ia and VI are the most prevalent among pregnant women in Malaysia (38, 39), while serotypes III predominate in China (16). According to Whitney et al. (2004), serotype V was most common among strains from Bangkok, Thailand accounting for 45.8% of strains from that site (40). Serotypes Ia, Ib, III and V are usually the most common in the United States, Europe (25). Serotype VI was also reported as a common colonizing serotype in women in Egypt and Taiwan (41, 35). Serotype II is common in Namibia and South Africa and is consistently higher in comparison to other areas (6). In Ghana, serotypes VII and IX are the most commonly isolated from pregnant women (42). The reasons for such varying prevalence of GBS serotypes might be explained the investigation of different geographical locations, source of the bacterial isolates, profile of the population being studied and period of time in these studies (6, 15, 43). Besides those, previous studies have shown that the distribution of GBS serotypes not only differs from one country to another but also between provinces within the same country, with changes in prevalence over time (18). Therefore, additional investigations should be made to clarify the prevalence and serotypes of group B *Streptococcus* in Vietnam.

## CONCLUSION

The findings of this study indicated that prevalence of GBS colonization in pregnant women in Nghe An province is 9.20% and comparable to rates observed among countries within the same region. The most prevalent serotypes were serotypes III and V. The high frequency of serotype VI was a notable feature of the strains from pregnant women in Vietnam.

## References

[1] Guo X-G Zhuang Y-R Wen J-Z Xie T-A Liu Y-L Zhu G-D Evaluation of the real-time fluorescence loop-mediated isothermal amplification assay for the detection of *Streptococcus agalactiae*. Biosci Rep 2019; 39:BSR20190383. 30988075 10.1042/BSR20190383 PMC6522725

[2] Poyart C Tazi A Réglier-Poupet H Billoët A Tavares N Raymond J Multiplex PCR assay for rapid and accurate capsular typing of group B streptococci. J Clin Microbiol 2007; 45: 1985–1988. 17376884 10.1128/JCM.00159-07 PMC1933079

[3] Wang P Ma Z Tong J Zhao R Shi W Yu S Serotype distribution, antimicrobial resistance, and molecular characterization of invasive group B *Streptococcus* isolates recovered from Chinese neonates. Int J Infect Dis 2015;37:115–118. 26141418 10.1016/j.ijid.2015.06.019

[4] Raabe VN Shane AL. Group B *Streptococcus (Streptococcus agalactiae)*. Microbiol Spectr 2019; 7: 10.1128/microbiolspec. 10.1128/microbiolspec.gpp3-0007-2018 PMC6432937 30900541

[5] Imperi M Pataracchia M Alfarone G Baldassarri L Orefici G Creti R. A multiplex PCR assay for the direct identification of the capsular type (Ia to IX) of *Streptococcus agalactiae*. J Microbiol Methods 2010; 80: 212–214. 19958797 10.1016/j.mimet.2009.11.010

[6] Mukesi M Iweriebor BC Obi LC Nwodo UU Moyo SR Okoh AI. Prevalence and capsular type distribution of *Streptococcus agalactiae* isolated from pregnant women in Namibia and South Africa. BMC Infect Dis 2019; 19:179. 30786878 10.1186/s12879-019-3809-6 PMC6383256

[7] Morozumi M Wajima T Takata M Iwata S Ubukata K. Molecular characteristics of group B *Streptococci* isolated from adults with invasive infections in Japan. J Clin Microbiol 2016; 54: 2695–2700. 27558182 10.1128/JCM.01183-16 PMC5078545

[8] Chen VL Avci FY Kasper DL. A maternal vaccine against group B *Streptococcus*: past, present, and future. Vaccine 2013;31:D13–D19. 23973342 10.1016/j.vaccine.2012.12.080 PMC3757342

[9] Verani JR McGee L Schrag SJ Division of Bacterial Diseases, National Center for Immunization and Respiratory Diseases, Centers for Disease Control and Prevention (CDC). Prevention of perinatal group B streptococcal disease-revised guidelines from CDC, 2010. MMWR Recomm Rep 2010;59:1–36. 21088663

[10] Dangor Z Lala SG Cutland CL Koen A Jose L Nakwa F Burden of invasive group B *Streptococcus* disease and early neurological sequelae in South African infants. PLoS One 2015; 10(4):e0123014. 25849416 10.1371/journal.pone.0123014 PMC4388823

[11] Skoff TH Farley MM Petit S Craig AS Schaffner W Gershman K Increasing burden of invasive group B streptococcal disease in nonpregnant adults, 1990–2007. Clin Infect Dis 2009; 49: 85–92. 19480572 10.1086/599369

[12] Ballard MS Schonheyder HC Knudsen JD Lyytikainen O Dryden M Kennedy KJ The changing epidemiology of group B *Streptococcus* bloodstream infection: a multi-national population-based assessment. Infect Dis (Lond) 2016; 48: 386–391. 26759190 10.3109/23744235.2015.1131330

[13] Slotved HC Kong F Lambertsen L Sauer S Gilbert GL. Serotype IX, a proposed new *Streptococcus agalactiae* serotype. J Clin Microbiol 2007; 45: 2929–2936. 17634306 10.1128/JCM.00117-07 PMC2045254

[14] Slotved HC Hoffmann S. Evaluation of procedures for typing of group B *Streptococcus*: a retrospective study. PeerJ 2017;5:e3105. 28321367 10.7717/peerj.3105 PMC5357338

[15] Wang P Tong J-j Ma X-h Song F-l Fan L Guo C-m Serotypes, antibiotic susceptibilities, and multi-locus sequence type profiles of *Streptococcus agalactiae* isolates circulating in Beijing, China. PLoS One 2015; 10(3):e0120035. 25781346 10.1371/journal.pone.0120035 PMC4363692

[16] Lu B Li D Cui Y Sui W Huang L Lu X. Epidemiology of Group B *Streptococcus* isolated from pregnant women in Beijing, China. Clin Microbiol Infect 2014; 20:O370–O373. 24118553 10.1111/1469-0691.12416

[17] Jannati E Roshani M Arzanlou M Habibzadeh S Rahimi G Shapuri R. Capsular serotype and antibiotic resistance of group B *streptococci* isolated from pregnant women in Ardabil, Iran. Iran J Microbiol 2012; 4: 130–135. 23066487 PMC3465538

[18] Africa CWJ Kaambo E. Group B *Streptococcus* Serotypes in Pregnant Women From the Western Cape Region of South Africa. Front Public Health 2018; 6:356. 30564566 10.3389/fpubh.2018.00356 PMC6288474

[19] Kim DH Min BJ Jung EJ Byun JM Jeong DH Lee KB Prevalence of group B *Streptococcus* colonization in pregnant women in a tertiary care center in Korea. Obstet Gynecol Sci 2018; 61: 575–583. 30254993 10.5468/ogs.2018.61.5.575 PMC6137023

[20] Villanueva-Uy ME Wongsiridej P Sangtawesin V Chiu V Tallo V Nazaire-Bermal N The burden of invasive neonatal Group B streptococcal (GBS) disease in Thailand and the Philippines. Southeast Asian J Trop Med Public Health 2015; 46: 728–737. 26867393

[21] Miller CS Handley KM Wrighton KC Frischkorn KR Thomas BC Banfield JF. Short-read assembly of full-length 16S amplicons reveals bacterial diversity in subsurface sediments. PLoS One 2013; 8(2):e56018. 23405248 10.1371/journal.pone.0056018 PMC3566076

[22] Russell NJ Seale AC O'Driscoll M O'Sullivan C Bianchi-Jassir F Gonzalez-Guarin J Maternal colonization with group B *Streptococcus* and serotype distribution worldwide: systematic review and meta-analyses. Clin Infect Dis 2017;65(suppl_2):S100–S111. 29117327 10.1093/cid/cix658 PMC5848259

[23] Stein RA. Group B *streptococci* in pregnancy: New perspectives for old challenges. Int J Clin Pract 2019;73(5):e13340. 30843310 10.1111/ijcp.13340

[24] Namavar Jahromi B Poorarian S Poorbarfehee S. The prevalence and adverse effects of group B streptococcal colonization during pregnancy. Arch Iran Med 2008;11:654–657. 18976037

[25] Ippolito DL James WA Tinnemore D Huang RR Dehart MJ Williams J Group B *Streptococcus* serotype prevalence in reproductive-age women at a tertiary care military medical center relative to global serotype distribution. BMC Infect Dis 2010;10:336. 21106080 10.1186/1471-2334-10-336 PMC3004907

[26] Hong JS Choi CW Park KU Kim SN Lee HJ Lee HR Genital group B *Streptococcus* carrier rate and serotype distribution in Korean pregnant women: implications for group B streptococcal disease in Korean neonates. J Perinat Med 2010; 38: 373–377. 20297897 10.1515/jpm.2010.050

[27] Nkembe NM Kamga HG Baiye WA Chafa AB Njotang PN. *Streptococcus agalactiae* prevalence and antimicrobial susceptibility pattern in vaginal and anorectal swabs of pregnant women at a tertiary hospital in Cameroon. BMC Res Notes 2018; 11:480. 30012198 10.1186/s13104-018-3589-x PMC6048704

[28] Sharmila V Joseph NM Arun Babu T Chaturvedula L Sistla S. Genital tract group B streptococcal colonization in pregnant women: a South Indian perspective. J Infect Dev Ctries 2011; 5: 592–595. 21841303 10.3855/jidc.1551

[29] Ali MM Woldeamanuel Y Woldetsadik DA Chaka TE Fenta DA Dinberu MT Prevalence of group B *Streptococcus* among pregnant women and newborns at Hawassa University comprehensive specialized hospital, Hawassa, Ethiopia. BMC Infect Dis 2019; 19:325. 30991960 10.1186/s12879-019-3859-9 PMC6469063

[30] El-Kersh TA Al-Nuaim LA Kharfy TA Al-Shammary FJ Al-Saleh SS Al-Zamel FA. Detection of genital colonization of group B streptococci during late pregnancy. Saudi Med J 2002; 23: 56–61. 11938365

[31] Wollheim C Sperhacke RD Fontana SKR Vanni AC Kato SK Araujo PR Group B *Streptococcus* detection in pregnant women via culture and PCR methods. Rev Soc Bras Med Trop 2017; 50: 179–183. 28562753 10.1590/0037-8682-0454-2016

[32] Rausch AV Gross A Droz S Bodmer T Surbek DV. Group B *Streptococcus* colonization in pregnancy: prevalence and prevention strategies of neonatal sepsis. J Perinat Med 2009; 37: 124–129. 19021455 10.1515/JPM.2009.020

[33] Ji W Zhang L. Colonization prevalence and antibiotic susceptibility of Group B *Streptococcus* in pregnant women over a 6-year period in Dongguan, China. PLoS One 2017; 12(8):e0183083. 28813477 10.1371/journal.pone.0183083 PMC5557540

[34] Song JY Lim JH Lim S Yong Z Seo HS. Progress toward a group B streptococcal vaccine. Hum Vaccin Immunother 2018;14:2669–2681. 29995578 10.1080/21645515.2018.1493326 PMC6314413

[35] Chien-Chung Lee Hsu Jen-Fu Janapatla Rajendra Prasad Chen Chyi-Liang Zhou Ying-Li Lien Reyin Cheng-Hsun. Clinical and microbiological characteristics of group B *Streptococcus* from pregnant women and diseased infants in intrapartum antibiotic prophylaxis Era in Taiwan. Sci Rep 2019; 9:13525. 31537886 10.1038/s41598-019-49977-2 PMC6753095

[36] Madrid L Seale AC Kohli-Lynch M Edmond KM Lawn JE Heath PT Infant group B Streptococcal disease incidence and serotypes worldwide: systematic review and meta-analyses. Clin Infect Dis 2017; 65(suppl_2):S160–S172. 29117326 10.1093/cid/cix656 PMC5850457

[37] Lin SM Zhi Y Ahn KB Lim S Seo HS. Status of group B streptococcal vaccine development. Clin Exp Vaccine Res 2018; 7: 76–81. 29399583 10.7774/cevr.2018.7.1.76 PMC5795048

[38] Karunakaran R Raja NS Hafeez A Puthucheary SD. Group B *Streptococcus* infection: epidemiology, serotypes, and antimicrobial susceptibility of selected isolates in the population beyond infancy (excluding females with genital tract- and pregnancy-related isolates) at the University Malaya Medical Centre, Kuala Lumpur. Jpn J Infect Dis 2009; 62: 192–194. 19468178

[39] Dhanoa A Karunakaran R Puthucheary SD. Serotype distribution and antibiotic susceptibility of group B *streptococci* in pregnant women. Epidemiol Infect 2010;138:979–981. 19889253 10.1017/S0950268809991105

[40] Whitney CG Daly S Limpongsanurak S Festin MR Thinn KK Chipato T The international infections in pregnancy study: group B streptococcal colonization in pregnant women. J Matern Fetal Neonatal Med 2004; 15: 267–274. 15280136 10.1080/14767050410001668617

[41] Shabayek S Abdalla S Abouzeid AM. Serotype and surface protein gene distribution of colonizing group B *Streptococcus* in women in Egypt. Epidemiol Infect 2014; 142: 208–210. 23561305 10.1017/S0950268813000848 PMC9152610

[42] Slotved H-C Dayie NTKD Banini JAN Frimodt-Møller N. Carriage and serotype distribution of *Streptococcus agalactiae* in third trimester pregnancy in southern Ghana. BMC Pregnancy Childbirth 2017; 17:238. 28732495 10.1186/s12884-017-1419-0 PMC5520380

[43] Botelho ACN Oliveira JG Damasco AP Santos KTB Ferreira AFM Rocha GT *Streptococcus agalactiae* carriage among pregnant women living in Rio de Janeiro, Brazil, over a period of eight years. PLoS One 2018; 13(5):e0196925. 29750801 10.1371/journal.pone.0196925 PMC5947911

